# Extranodal natural killer/T‐cell lymphoma in an 11‐year‐old child

**DOI:** 10.1002/ccr3.3474

**Published:** 2020-11-03

**Authors:** Takeshi Ninchoji, Junya Fujimura, Suguru Uemura, Nobuyuki Yamamoto, Kandai Nozu, Kazumoto Iijima

**Affiliations:** ^1^ Kobe Graduate University of Medicine, Pediatrics Kobe Japan

**Keywords:** extranodal natural killer/T‐cell lymphoma, hemophagocytic syndrome, SMILE therapy

## Abstract

Extranodal natural killer/T‐cell lymphoma (ENKTL) is difficult to identify and diagnose appropriately. Positron emission tomography imaging is a crucial method that leads to precise diagnosis. A proper regimen including stem cell transplantation would possibly improve prognosis of advanced ENKTL.

## Graphical abstract

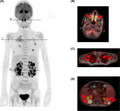

## 1 CASE

**FIGURE 1:**
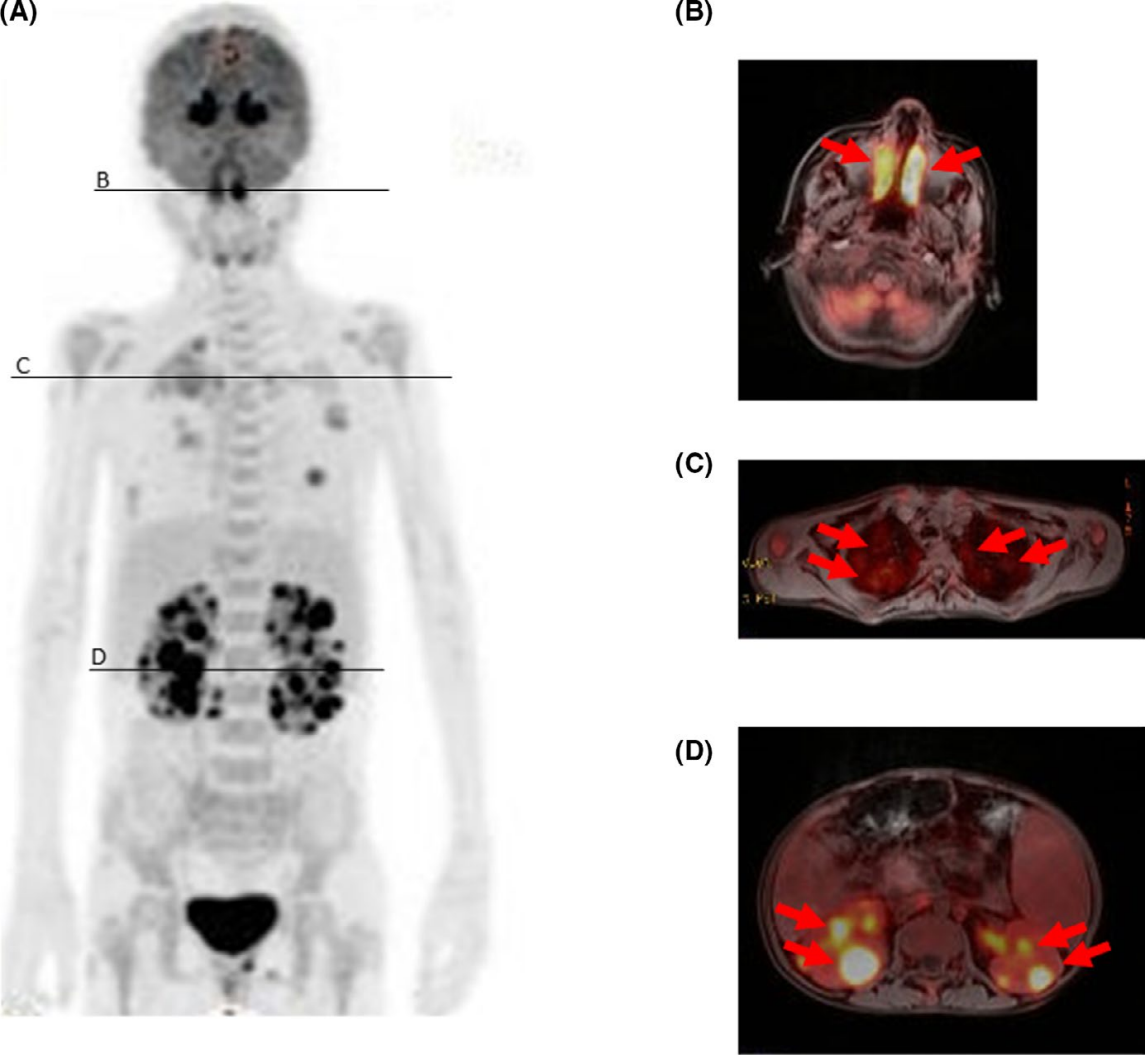
PET‐MRI images. A, Whole scan showed abnormal accumulations in the sinusoid, lungs, and kidney. B‐D, Coronal images of each organ. B, sinusoid; C. lungs; D, kidney

An 11‐year‐old girl presented with visual deterioration and cough for several days. An ophthalmologist diagnosed her with uveitis. Computed tomography showed infiltrates in the lung field. She developed fever (38.5°C) on the day of admission. We initially diagnosed her with mycoplasma infection based on an elevated IgM antibody titer of 1:320 and administered her antibiotics. However, her fever persisted for 7 days, and laboratory findings met the HLH‐2004 diagnostic criteria.^1^ Considering other systemic diseases, we performed positron emission tomography‐magnetic resonance imaging (PET‐MRI) that demonstrated several abnormal accumulations (Figure 1). Pathologic investigation of sinusoid, lung, and kidney biopsies showed diffusely increased lymphoid cells with atypical nuclear features surrounded by necrotic background. Immunochemistry showed positivity for CD3, 4, 7, 8, and 56, and negativity for CD19 and 20 with positivity for EBV‐encoded RNA in situ hybridization. The lack of a history of mosquito allergy and infectious mononucleosis‐like syndrome led us to establish a diagnosis of extranodal natural killer/T‐cell lymphoma, nasal type (ENKTL), stage IV.^2^ The patient was treated with dexamethasone, methotrexate, ifosfamide, L‐asparaginase, and etoposide (SMILE) for six courses following umbilical cord blood stem cell transplantation (SCT) and has achieved complete remission for 3 years.

Extranodal natural killer/T‐cell lymphoma is rare in children. Previous reports have documented multiple organ involvement, including the nasal cavity, skin, gastrointestinal tract, liver, and lungs. However, a few cases have reported involvement of the eyes and kidneys. The most distinctive symptom was visual deterioration. Since ENKTL is difficult to identify, delayed diagnosis or misdiagnosis can occur. In this case, we initially diagnosed her with an infectious disease until PET‐MRI was performed, which was crucial for diagnosis.

Regarding treatment, ENKTL stage IV with hemophagocytic syndrome has been reported to have the worst prognosis. A strong regimen with SMILE in addition to SCT was recommended in adult‐onset advanced ENKTL.^3^, ^4^ We administered the same regimen in this child, which possibly improved the prognosis and proved successful.

## CONFLICT OF INTEREST

Kandai Nozu has received consulting fees from Kyowa Kirin Co., Ltd.; and lecture fees from Kyowa Kirin Co., Ltd., Novartis Pharmaceuticals Corporation, and Chugai Pharmaceutical Co., Ltd. Kazumoto Iijima has received grant support from Daiichi Sankyo Co., Ltd.; consulting fees from Kyowa Kirin Co., Ltd., and Boehringer Ingelheim; and lecture fees from Kyowa Kirin Co., Ltd., Chugai Pharmaceutical Co., Ltd., Takeda Pharmaceutical Company, Integrated Development Associates, and Novartis Pharmaceuticals Corporation. The other authors declare that no conflicts of interest exist.

## AUTHOR CONTRIBUTIONS

TN: made substantial contribution to the preparation of this manuscript and approved the final version for submission. JF, SU, NY, KN, and KI: did the literature search, revised the manuscript for critically important intellectual content, and approved the final version for submission.

## ETHICAL APPROVAL

All procedures performed in studies involving human participants were in accordance with the ethical standard of the Russian Federation with the 1964 Helsinki declarations and its later amendments or comparable ethical standard. Enrolled patients provided written informed consent. All preparation and practice are officially certificated (B190207 Kobe university).

## INFORMED CONSENT

Informed consent was obtained for publication of this clinical images.

## Data Availability

In accordance with the DFG guidelines on the Handling of Research Data, we made all images as TIFF format available upon request.
